# Ten Simple Rules for Reproducible Computational Research

**DOI:** 10.1371/journal.pcbi.1003285

**Published:** 2013-10-24

**Authors:** Geir Kjetil Sandve, Anton Nekrutenko, James Taylor, Eivind Hovig

**Affiliations:** 1 Department of Informatics, University of Oslo, Blindern, Oslo, Norway; 2 Centre for Cancer Biomedicine, University of Oslo, Blindern, Oslo, Norway; 3 Department of Biochemistry and Molecular Biology and The Huck Institutes for the Life Sciences, Penn State University, University Park, Pennsylvania, United States of America; 4 Department of Biology and Department of Mathematics and Computer Science, Emory University, Atlanta, Georgia, United States of America; 5 Department of Tumor Biology, Institute for Cancer Research, The Norwegian Radium Hospital, Oslo University Hospital, Montebello, Oslo, Norway; 6 Institute for Medical Informatics, The Norwegian Radium Hospital, Oslo University Hospital, Montebello, Oslo, Norway; University of California San Diego, United States of America

Replication is the cornerstone of a cumulative science [1]. However, new tools and technologies, massive amounts of data, interdisciplinary approaches, and the complexity of the questions being asked are complicating replication efforts, as are increased pressures on scientists to advance their research [2]. As full replication of studies on independently collected data is often not feasible, there has recently been a call for reproducible research as an attainable minimum standard for assessing the value of scientific claims [3]. This requires that papers in experimental science describe the results and provide a sufficiently clear protocol to allow successful repetition and extension of analyses based on original data [4].

The importance of replication and reproducibility has recently been exemplified through studies showing that scientific papers commonly leave out experimental details essential for reproduction [5], studies showing difficulties with replicating published experimental results [6], an increase in retracted papers [7], and through a high number of failing clinical trials [8], [9]. This has led to discussions on how individual researchers, institutions, funding bodies, and journals can establish routines that increase transparency and reproducibility. In order to foster such aspects, it has been suggested that the scientific community needs to develop a “culture of reproducibility” for computational science, and to require it for published claims [3].

We want to emphasize that reproducibility is not only a moral responsibility with respect to the scientific field, but that a lack of reproducibility can also be a burden for you as an individual researcher. As an example, a good practice of reproducibility is necessary in order to allow previously developed methodology to be effectively applied on new data, or to allow reuse of code and results for new projects. In other words, good habits of reproducibility may actually turn out to be a time-saver in the longer run.

We further note that reproducibility is just as much about the habits that ensure reproducible research as the technologies that can make these processes efficient and realistic. Each of the following ten rules captures a specific aspect of reproducibility, and discusses what is needed in terms of information handling and tracking of procedures. If you are taking a bare-bones approach to bioinformatics analysis, i.e., running various custom scripts from the command line, you will probably need to handle each rule explicitly. If you are instead performing your analyses through an integrated framework (such as GenePattern [10], Galaxy [11], LONI pipeline [12], or Taverna [13]), the system may already provide full or partial support for most of the rules. What is needed on your part is then merely the knowledge of how to exploit these existing possibilities.

In a pragmatic setting, with publication pressure and deadlines, one may face the need to make a trade-off between the ideals of reproducibility and the need to get the research out while it is still relevant. This trade-off becomes more important when considering that a large part of the analyses being tried out never end up yielding any results. However, frequently one will, with the wisdom of hindsight, contemplate the missed opportunity to ensure reproducibility, as it may already be too late to take the necessary notes from memory (or at least much more difficult than to do it while underway). We believe that the rewards of reproducibility will compensate for the risk of having spent valuable time developing an annotated catalog of analyses that turned out as blind alleys.

As a minimal requirement, you should at least be able to reproduce the results yourself. This would satisfy the most basic requirements of sound research, allowing any substantial future questioning of the research to be met with a precise explanation. Although it may sound like a very weak requirement, even this level of reproducibility will often require a certain level of care in order to be met. There will for a given analysis be an exponential number of possible combinations of software versions, parameter values, pre-processing steps, and so on, meaning that a failure to take notes may make exact reproduction essentially impossible.

With this basic level of reproducibility in place, there is much more that can be wished for. An obvious extension is to go from a level where you can reproduce results in case of a critical situation to a level where you can practically and routinely reuse your previous work and increase your productivity. A second extension is to ensure that peers have a practical possibility of reproducing your results, which can lead to increased trust in, interest for, and citations of your work [6], [14].

We here present ten simple rules for reproducibility of computational research. These rules can be at your disposal for whenever you want to make your research more accessible—be it for peers or for your future self.

## Rule 1: For Every Result, Keep Track of How It Was Produced

Whenever a result may be of potential interest, keep track of how it was produced. When doing this, one will frequently find that getting from raw data to the final result involves many interrelated steps (single commands, scripts, programs). We refer to such a sequence of steps, whether it is automated or performed manually, as an analysis workflow. While the essential part of an analysis is often represented by only one of the steps, the full sequence of pre- and post-processing steps are often critical in order to reach the achieved result. For every involved step, you should ensure that every detail that may influence the execution of the step is recorded. If the step is performed by a computer program, the critical details include the name and version of the program, as well as the exact parameters and inputs that were used.

Although manually noting the precise sequence of steps taken allows for an analysis to be reproduced, the documentation can easily get out of sync with how the analysis was really performed in its final version. By instead specifying the full analysis workflow in a form that allows for direct execution, one can ensure that the specification matches the analysis that was (subsequently) performed, and that the analysis can be reproduced by yourself or others in an automated way. Such executable descriptions [10] might come in the form of simple shell scripts or makefiles [15], [16] at the command line, or in the form of stored workflows in a workflow management system [10], [11], [13], [17], [18].

As a minimum, you should at least record sufficient details on programs, parameters, and manual procedures to allow yourself, in a year or so, to approximately reproduce the results.

## Rule 2: Avoid Manual Data Manipulation Steps

Whenever possible, rely on the execution of programs instead of manual procedures to modify data. Such manual procedures are not only inefficient and error-prone, they are also difficult to reproduce. If working at the UNIX command line, manual modification of files can usually be replaced by the use of standard UNIX commands or small custom scripts. If working with integrated frameworks, there will typically be a quite rich collection of components for data manipulation. As an example, manual tweaking of data files to attain format compatibility should be replaced by format converters that can be reenacted and included into executable workflows. Other manual operations like the use of copy and paste between documents should also be avoided. If manual operations cannot be avoided, you should as a minimum note down which data files were modified or moved, and for what purpose.

## Rule 3: Archive the Exact Versions of All External Programs Used

In order to exactly reproduce a given result, it may be necessary to use programs in the exact versions used originally. Also, as both input and output formats may change between versions, a newer version of a program may not even run without modifying its inputs. Even having noted which version was used of a given program, it is not always trivial to get hold of a program in anything but the current version. Archiving the exact versions of programs actually used may thus save a lot of hassle at later stages. In some cases, all that is needed is to store a single executable or source code file. In other cases, a given program may again have specific requirements to other installed programs/packages, or dependencies to specific operating system components. To ensure future availability, the only viable solution may then be to store a full virtual machine image of the operating system and program. As a minimum, you should note the exact names and versions of the main programs you use.

## Rule 4: Version Control All Custom Scripts

Even the slightest change to a computer program can have large intended or unintended consequences. When a continually developed piece of code (typically a small script) has been used to generate a certain result, only that exact state of the script may be able to produce that exact output, even given the same input data and parameters. As also discussed for rules 3 and 6, exact reproduction of results may in certain situations be essential. If computer code is not systematically archived along its evolution, backtracking to a code state that gave a certain result may be a hopeless task. This can cast doubt on previous results, as it may be impossible to know if they were partly the result of a bug or otherwise unfortunate behavior.

The standard solution to track evolution of code is to use a version control system [15], such as Subversion, Git, or Mercurial. These systems are relatively easy to set up and use, and may be used to systematically store the state of the code throughout development at any desired time granularity.

As a minimum, you should archive copies of your scripts from time to time, so that you keep a rough record of the various states the code has taken during development.

## Rule 5: Record All Intermediate Results, When Possible in Standardized Formats

In principle, as long as the full process used to produce a given result is tracked, all intermediate data can also be regenerated. In practice, having easily accessible intermediate results may be of great value. Quickly browsing through intermediate results can reveal discrepancies toward what is assumed, and can in this way uncover bugs or faulty interpretations that are not apparent in the final results. Secondly, it more directly reveals consequences of alternative programs and parameter choices at individual steps. Thirdly, when the full process is not readily executable, it allows parts of the process to be rerun. Fourthly, when reproducing results, it allows any experienced inconsistencies to be tracked to the steps where the problems arise. Fifth, it allows critical examination of the full process behind a result, without the need to have all executables operational. When possible, store such intermediate results in standardized formats. As a minimum, archive any intermediate result files that are produced when running an analysis (as long as the required storage space is not prohibitive).

## Rule 6: For Analyses That Include Randomness, Note Underlying Random Seeds

Many analyses and predictions include some element of randomness, meaning the same program will typically give slightly different results every time it is executed (even when receiving identical inputs and parameters). However, given the same initial seed, all random numbers used in an analysis will be equal, thus giving identical results every time it is run. There is a large difference between observing that a result has been reproduced exactly or only approximately. While achieving equal results is a strong indication that a procedure has been reproduced exactly, it is often hard to conclude anything when achieving only approximately equal results. For analyses that involve random numbers, this means that the random seed should be recorded. This allows results to be reproduced exactly by providing the same seed to the random number generator in future runs. As a minimum, you should note which analysis steps involve randomness, so that a certain level of discrepancy can be anticipated when reproducing the results.

## Rule 7: Always Store Raw Data behind Plots

From the time a figure is first generated to it being part of a published article, it is often modified several times. In some cases, such modifications are merely visual adjustments to improve readability, or to ensure visual consistency between figures. If raw data behind figures are stored in a systematic manner, so as to allow raw data for a given figure to be easily retrieved, one can simply modify the plotting procedure, instead of having to redo the whole analysis. An additional advantage of this is that if one really wants to read fine values in a figure, one can consult the raw numbers. In cases where plotting involves more than a direct visualization of underlying numbers, it can be useful to store both the underlying data and the processed values that are directly visualized. An example of this is the plotting of histograms, where both the values before binning (original data) and the counts per bin (heights of visualized bars) could be stored. When plotting is performed using a command-based system like R, it is convenient to also store the code used to make the plot. One can then apply slight modifications to these commands, instead of having to specify the plot from scratch. As a minimum, one should note which data formed the basis of a given plot and how this data could be reconstructed.

## Rule 8: Generate Hierarchical Analysis Output, Allowing Layers of Increasing Detail to Be Inspected

The final results that make it to an article, be it plots or tables, often represent highly summarized data. For instance, each value along a curve may in turn represent averages from an underlying distribution. In order to validate and fully understand the main result, it is often useful to inspect the detailed values underlying the summaries. A common but impractical way of doing this is to incorporate various debug outputs in the source code of scripts and programs. When the storage context allows, it is better to simply incorporate permanent output of all underlying data when a main result is generated, using a systematic naming convention to allow the full data underlying a given summarized value to be easily found. We find hypertext (i.e., html file output) to be particularly useful for this purpose. This allows summarized results to be generated along with links that can be very conveniently followed (by simply clicking) to the full data underlying each summarized value. When working with summarized results, you should as a minimum at least once generate, inspect, and validate the detailed values underlying the summaries.

## Rule 9: Connect Textual Statements to Underlying Results

Throughout a typical research project, a range of different analyses are tried and interpretation of the results made. Although the results of analyses and their corresponding textual interpretations are clearly interconnected at the conceptual level, they tend to live quite separate lives in their representations: results usually live on a data area on a server or personal computer, while interpretations live in text documents in the form of personal notes or emails to collaborators. Such textual interpretations are not generally mere shadows of the results—they often involve viewing the results in light of other theories and results. As such, they carry extra information, while at the same time having their necessary support in a given result.

If you want to reevaluate your previous interpretations, or allow peers to make their own assessment of claims you make in a scientific paper, you will have to connect a given textual statement (interpretation, claim, conclusion) to the precise results underlying the statement. Making this connection when it is needed may be difficult and error-prone, as it may be hard to locate the exact result underlying and supporting the statement from a large pool of different analyses with various versions.

To allow efficient retrieval of details behind textual statements, we suggest that statements are connected to underlying results already from the time the statements are initially formulated (for instance in notes or emails). Such a connection can for instance be a simple file path to detailed results, or the ID of a result in an analysis framework, included within the text itself. For an even tighter integration, there are tools available to help integrate reproducible analyses directly into textual documents, such as Sweave [19], the GenePattern Word add-in [4], and Galaxy Pages [20]. These solutions can also subsequently be used in connection with publications, as discussed in the next rule.

As a minimum, you should provide enough details along with your textual interpretations so as to allow the exact underlying results, or at least some related results, to be tracked down in the future.

## Rule 10: Provide Public Access to Scripts, Runs, and Results

Last, but not least, all input data, scripts, versions, parameters, and intermediate results should be made publicly and easily accessible. Various solutions have now become available to make data sharing more convenient, standardized, and accessible in particular domains, such as for gene expression data [21]–[23]. Most journals allow articles to be supplemented with online material, and some journals have initiated further efforts for making data and code more integrated with publications [3], [24]. As a minimum, you should submit the main data and source code as supplementary material, and be prepared to respond to any requests for further data or methodology details by peers.

Making reproducibility of your work by peers a realistic possibility sends a strong signal of quality, trustworthiness, and transparency. This could increase the quality and speed of the reviewing process on your work, the chances of your work getting published, and the chances of your work being taken further and cited by other researchers after publication [25].
